# Wheat Dwarf Virus as a Modulator of Multi‐Stress Responses in Wheat

**DOI:** 10.1111/ppl.70931

**Published:** 2026-05-13

**Authors:** Jana Asszonyi

**Affiliations:** ^1^ Department of Crop Science, Breeding and Plant Protection, Faculty of AgriSciences Mendel University in Brno Brno Czech Republic

**Keywords:** abiotic and biotic stress, phytohormone regulation, plant–virus interactions, redox homeostasis, wheat dwarf virus

## Abstract

Wheat dwarf virus (WDV) is an emerging constraint to cereal production whose epidemiological significance has intensified under climate change. Rising temperatures, extended vector activity, and the expansion of *Psammotettix alienus* into new regions have increased both the frequency and severity of WDV outbreaks. Beyond its direct effects on plant development, WDV acts as a powerful regulator of host physiology, functioning as a host signalling hub that reprograms hormonal signalling, alters source‐sink relationships, disrupts redox homeostasis, and modulates responses to both abiotic and biotic stress. Recent molecular studies have revealed how viral proteins manipulate the cell cycle, transcriptional machinery, and RNA silencing pathways to optimise viral replication while attenuating defence responses. These processes intersect with core stress‐response networks, particularly those governed by abscisic acid, gibberellins, cytokinins, and auxin, positioning WDV as a model system for investigating hormonal crosstalk under combined stress. Despite advances in genomics, transcriptomics, and vector biology, major knowledge gaps persist regarding WDV interactions with co‐occurring fungal pathogens, its impact on the plant microbiome, and its role in shaping cereal resilience under drought, heat, or nutrient limitations. This review synthesises current understanding of WDV biology from the molecular to the ecological scale, highlights mechanisms underpinning stress integration, and outlines future research priorities essential for developing sustainable management strategies in a changing climate.

## 1 Introduction

Wheat dwarf virus (WDV), first identified in the former Czechoslovak Republic (Vacke 1961), has become an increasing threat to cereal production worldwide under conditions of ongoing climatic change, infecting a broad range of *Poaceae* species and causing substantial yield losses in wheat ( *Triticum aestivum* ) and barley ( *Hordeum vulgare* ) (Abt et al. 2020; Wei et al. 2024). Transmission occurs via the leafhopper *Psammotettix alienus* in a persistent, non‐propagative manner, and recent climatic changes have strongly influenced the ecology of this vector. Warmer temperatures and extended frost‐free periods have prolonged vector activity and enabled its expansion into new geographical areas across Europe, Africa, and Asia, thereby increasing the incidence and severity of WDV outbreaks (Ekzayez et al. 2011; Pfrieme et al. 2023; Xie et al. 2007). Climate projections suggest that the distribution and population dynamics of vector‐borne cereal viruses will continue to shift, with region‐specific consequences depending on vector ecology and environmental conditions (Akbaş et al. 2022).

Simultaneously, rising global temperatures, recurrent drought, and altered precipitation regimes are intensifying pressure from both biotic and abiotic stressors. These conditions have increased the prevalence of viral pathogens in cereals, including WDV, and facilitated the spread of previously sporadic or locally restricted infections (İlbağı and Çıtır 2024). Vector‐borne viruses and their insect vectors are now recognised as major contributors to yield instability in wheat production systems, and their importance is projected to increase in northern and central European regions under future climate scenarios (Kapoor 2022). In many production areas, WDV co‐occurs with additional viral, fungal, and environmental stresses, making it essential to examine its impact not as an isolated pathogen but as part of a broader network of interacting stress factors.

Although early research focused primarily on symptomatology and diagnostic detection, recent advances in molecular virology, transcriptomics, and plant stress physiology have reshaped our understanding of WDV–host interactions. WDV is now recognised as a potent modulator of hormonal signalling, transcriptional regulation, photosynthetic function, cell cycle control, and redox homeostasis (Liu, Liu, et al. 2020; Jiang and Zhou 2023). These processes underpin both viral replication and the characteristic dwarfing and chlorosis symptoms observed in infected wheat (Lindblad and Sigvald 2004; Liu, Liu, et al. 2020). At the same time, the complexity of plant antiviral defence, which includes RNA silencing, autophagy, ubiquitination, chromatin modifications, miRNA‐mediated regulation, and hormone‐dependent signalling, illustrates the dynamic interplay between viral effectors and host immune pathways (Hanley‐Bowdoin et al. 2013; Gupta et al. 2021).

Despite these advances, critical gaps remain. Studies addressing WDV interactions with other pathogens, particularly major fungal diseases such as *Fusarium* spp., *Zymoseptoria* spp., and *Puccinia* spp., are virtually absent. This is notable given that evidence from related cereal viruses indicates strong potential for synergistic or antagonistic effects (Koch and Huth 1997; Liu and Buchenauer 2005). Similarly, the consequences of WDV infection for the plant microbiome remain poorly understood, although root‐associated microbial communities are increasingly recognised for their essential roles in stress resilience (Ma et al. 2020; Ma et al. 2019). Moreover, transcriptomic evidence suggests that WDV profoundly rewires hormonal networks. However, the mechanistic implications of these disturbances for stress integration and susceptibility to co‐infections remain unresolved (Liu, Liu, et al. 2020).

Resistance of wheat to WDV is under complex genetic control. Quantitative trait loci Qwdv.ifa‐6A and Qwdv.ifa‐1B have been identified, together accounting for up to 70% of phenotypic variance (Buerstmayr and Buerstmayr 2023), and additional resistance sources have been reported in *Aegilops* species and legacy wheat cultivars (Benkovics et al. 2010; Nygren et al. 2015). Despite these advances, the mechanistic basis of WDV tolerance remains poorly resolved, particularly regarding the coordination between morphological traits, physiological performance, and transcriptional defence responses. An integrative framework combining field experiments with morphological, physiological, and molecular analyses is therefore essential to elucidate WDV‐induced effects in winter wheat and to identify resistant genotypes with potential value for breeding.

## 2 A Framework for Understanding WDV as a Host Signalling Hub

Here, WDV is considered a host signalling hub reweighting growth, defence, and acclimation. In this view, infection is not limited to direct viral damage or isolated pathway perturbations. Instead, WDV alters the balance among hormonal signalling, ROS dynamics, RNA silencing, source‐sink relationships, and cell‐cycle control, thereby reshaping how wheat responds to abiotic and biotic stresses.

This framework offers three main conceptual advances. First, it explains why WDV effects under combined stress are unlikely to be additive, because drought, heat, nutrient limitation, and co‐infecting pathogens converge on many of the same regulatory nodes. Second, it predicts strong context dependence, in which genotype, developmental stage, and infection order influence whether reprogramming promotes transient acclimation or heightened susceptibility. Third, it shifts the interpretation of WDV symptoms from isolated disease traits to emergent outputs of a perturbed signalling system.

The following sections apply this framework to reinterpret WDV genome architecture, replication strategy, symptom development, hormonal crosstalk, and interactions with other stressors as interconnected components of a shared host‐response network.

## 3 WDV and Abiotic Stress Interactions

The host‐signalling‐hub framework is particularly useful under combined stress scenarios, because it explains why WDV effects under drought, heat, or nutrient limitation are unlikely to be simple sums of independent stress responses. Instead, viral and abiotic cues converge on overlapping regulatory nodes.

Experimental findings reveal substantial overlap between plant responses to biotic stress and to heat, drought, and salinity, with phytohormone‐mediated crosstalk shaping adaptation to combined stress conditions (Checker et al. 2018; Rahman et al. 2021; Westwood et al. 2013).

Despite these insights, the interaction between plant viruses and abiotic stresses remains incompletely understood. Recent observations show that virus‐infected plants sometimes display increased tolerance to drought and cold, whereas elevated temperatures can exacerbate viral virulence and disease severity (Prasad et al. 2022). Combined viral infection and cold stress in wheat enhance physiological and biochemical defence responses, including changes in redox balance, accumulation of H_2_O_2_ and lipid peroxidation products, and induction of antioxidant enzymes and PR proteins, which may support yield stability under stress (Molodchenkova et al. 2024).

Plant–virus interactions, including those involving WDV, represent useful models for investigating overlap between biotic and abiotic stress responses. RNA silencing links antiviral defence to stress‐regulatory pathways (Rahman et al. 2021; Ma et al. 2020, 2019).

**FIGURE 1:**
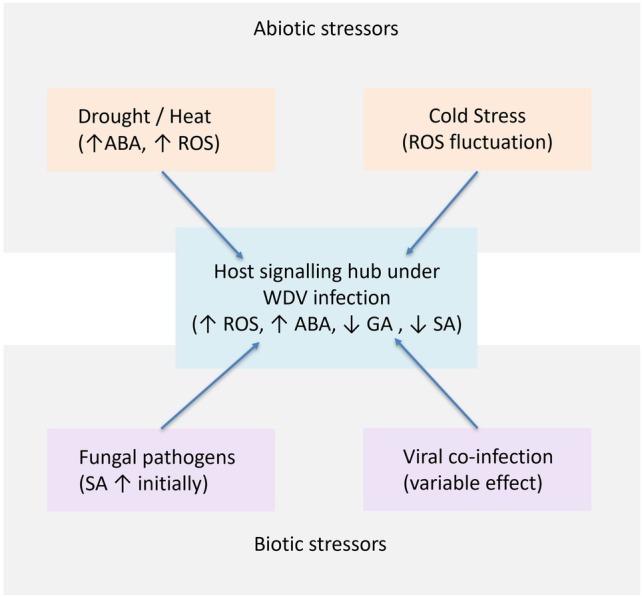
Convergence of multiple stress pathways on the WDV‐altered host signalling hub.

This integration of abiotic and biotic cues at the WDV‐altered host signalling hub provides a mechanistic basis for understanding the complex symptomology and stress outcomes observed in the field and reinforces the view of WDV as a central modulator of multi‐stress responses (Figure 1).

Abiotic stress outcomes during WDV infection are unlikely to be additive because drought, heat, and nutrient limitation converge on many of the same hub nodes targeted by the virus, including ABA signalling, redox control, source‐sink dynamics, and RNA silencing. In this context, WDV can shift the plant into a stress‐mimicking physiological state that may transiently increase tolerance to particular stresses through stomatal closure and stress acclimation, while simultaneously reducing growth capacity and altering defence prioritisation. The hub framework therefore predicts strong context dependence. The net effect of WDV combined with abiotic stress should vary with stress timing and intensity and with genotype‐specific hormonal coordination. Under this framework, the critical question is not whether WDV worsens or alleviates abiotic stress in general, but under which combinations of timing, intensity, and genotype the virus redirects host regulation toward transient acclimation or increased vulnerability.

## 4 WDV and Biotic Stress Interactions

Biotic interactions strongly test the hub framework because co‐infecting pathogens often engage the same hormonal, redox, and defence nodes altered by WDV. This makes infection order, developmental stage, and defence prioritisation central variables in determining outcome.

### 4.1 Synergistic or Antagonistic Effects With Other Viruses

Mixed viral infections are frequently observed in cereals and may include Wheat dwarf virus (WDV) in association with other pathogens such as Barley yellow dwarf virus (BYDV), Wheat streak mosaic virus (WSMV), Barley mild mosaic virus (BaMMV), Soil‐borne wheat mosaic virus (SBWMV), and Wheat spindle streak mosaic virus (WSSMV) (Achon et al. 2006; Choudhury et al. 2017; Deb and Anderson 2008; Trzmiel and Hasiów‐Jaroszewska 2023). Evidence from co‐infection studies shows that virus–virus interactions are highly context dependent and can be antagonistic or synergistic, with outcomes influenced by infection order, plant age, and replication dynamics. In wheat, asymmetric interactions between WDV and BYDV‐PAV have been reported, where WDV can facilitate BYDV‐PAV accumulation under sequential inoculation, while BYDV‐PAV can reduce WDV transmission and accumulation in other co‐infection scenarios (Armand et al. 2023). No consistent trend in virus titre was observed for any of the viruses, except for WSMV, which showed an approximately fivefold reduction in titre when co‐infected with either BYDV or WDV, compared to WSMV infection alone. This may suggest the influence of WDV in mixed infections (Jarošová et al. 2018). Under the hub framework, reductions in titre during co‐infection may reflect competition for host resources or altered antiviral signalling. Wheat can be co‐infected by WSMV, TriMV, BMV, and BSMV, leading to complex interactions. BMV suppressed BSMV accumulation, while WSMV–TriMV co‐infection caused strong synergism with elevated titre. Triple infections enhanced disease severity, and quadripartite infections led to lethal synergism despite reduced BSMV levels. These findings show that virus–virus interactions in wheat are highly complex and not always linked to increased virus titre (Tatineni et al. 2022).

### 4.2 Interaction With Fungal Pathogens

Wheat plants infected with virus pathogen (BYDV) exhibited increased Fusarium Head Blight (FHB) severity and elevated DON mycotoxin levels, suggesting interaction between the virus and the fungus (Liu and Buchenauer 2005). Specifically, BYDV infection increases wheat susceptibility to *Fusarium* spp., particularly when viral infection occurs at later growth stages, resulting in more severe Fusarium Head Blight and higher DON accumulation. BYDV at the early tillering stage (EC 25/35) and later challenged with *Fusarium culmorum* at EC 55/65 showed less yield loss than those subjected to simultaneous infections at flowering, indicating that BYDV infection during later developmental stages enhances wheat susceptibility to *F. culmorum*. This demonstrates that the timing of viral infection influences subsequent fungal colonization and mycotoxin contamination, thereby intensifying yield and quality losses (Koch and Huth 1997). Within the hub framework, these timing effects may reflect stage‐dependent shifts in hormonal coordination and defence prioritisation, which can influence anti‐fungal immunity. In this context, the identification of resistance genes in certain wild wheat relatives (*Aegilops*) that provide protection against fungal pathogens as well as other pathogens, including viruses, supports the hypothesis of overlapping resistance pathways and convergence on shared defence signalling nodes, as conceptualised in the WDV‐altered host signalling hub (Figure 1) (Nygren et al. 2015; Schneider et al. 2008).

Biotic interactions provide a high‐impact test of the hub concept because co‐infecting pathogens often target overlapping defence and hormone nodes. The observed context dependence in virus–virus interactions, combined with the near absence of WDV‐fungus studies, suggests that outcomes may hinge on infection order and on whether WDV‐driven ABA/SA/ROS reweighting suppresses or redirects anti‐fungal immunity. Extending this logic to microbiome effects, WDV‐driven changes in hormones and carbon allocation could plausibly reshape rhizosphere recruitment and systemic resistance, positioning the microbiome as an underexplored but potentially tractable leverage point for multi‐stress management.

## 5 Viral Functional Modules That Feed Host Reprogramming

Rather than treating WDV genome architecture as descriptive background alone, it is useful to view the viral genome as a set of functional modules that determine where and how the virus interfaces with core host regulatory systems. In the signalling‐hub framework, these modules represent the mechanistic entry points through which WDV reprograms wheat physiology.

**FIGURE 2:**
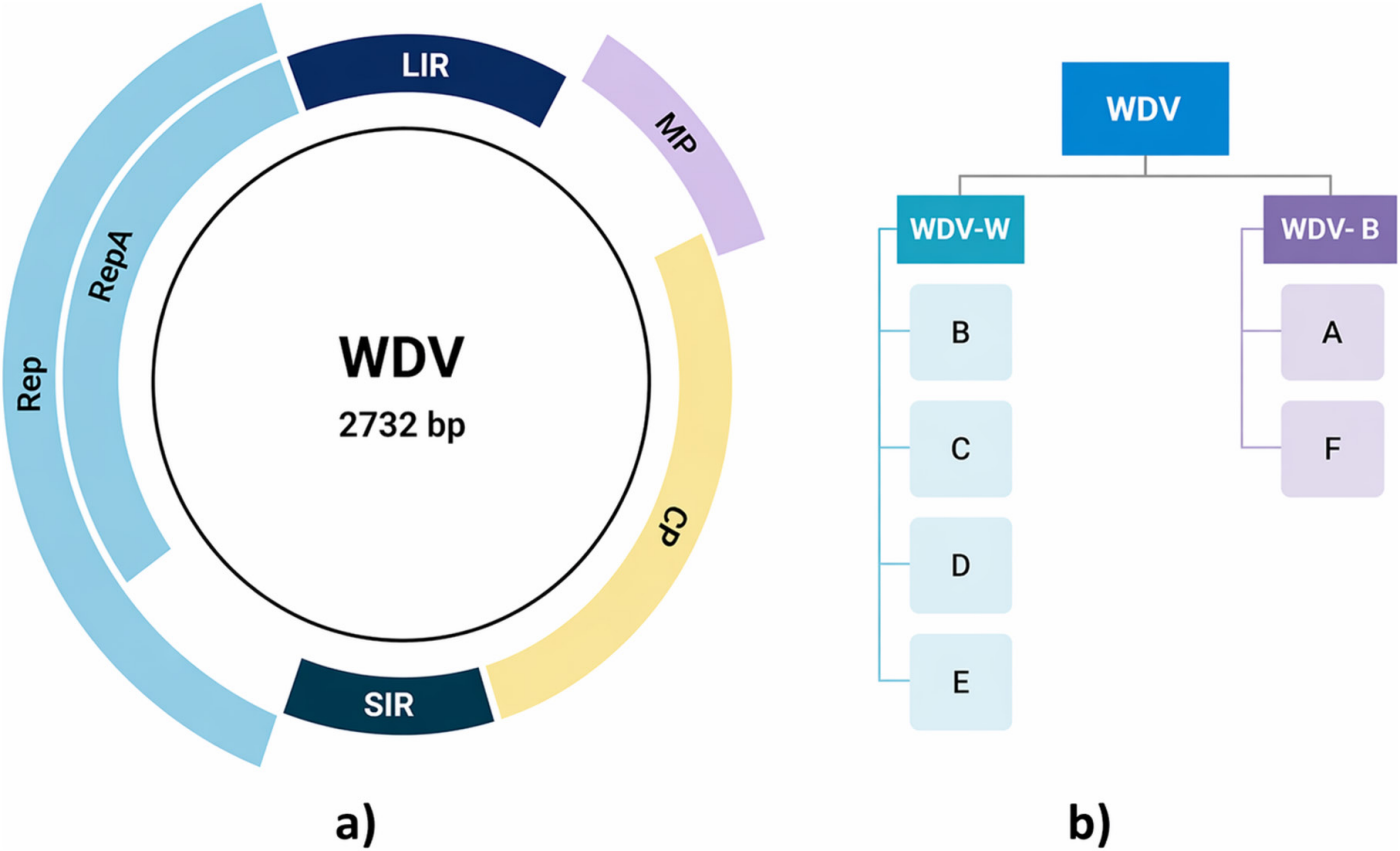
(a) Genomic structure, modified from Çelik and Ferhan Morca (2021), (b) Strain Classification of Wheat Dwarf Virus (WDV), text source: (Wu et al. 2015). Unlike many Begomoviruses (genus Begomovirus), WDV (genus Mastrevirus) does not encode a distinct replication enhancer (REn/C3): Rep/RepA are expressed via alternative splicing.

Wheat dwarf virus (WDV), a member of the genus *Mastrevirus* (family *Geminiviridae*), possesses a circular, monopartite single‐stranded DNA genome approximately 2.7 kb in length. Despite its compact size, the genome encodes four multifunctional proteins arranged bidirectionally around a long (LIR) and short (SIR) intergenic region that houses the origin of replication and essential transcriptional regulatory motifs. The viral strand encodes the movement protein (MP) and coat protein (CP), while the complementary strand encodes two replication‐associated proteins, Rep and RepA, generated through alternative splicing of a single open reading frame (Gutierrez 1999; Kvarnheden et al. 2002; Tóbiás et al. 2011). This streamlined genetic layout reflects the virus's dependence on host nuclear processes for replication, transcription, and intercellular movement (Figure 2a).

WDV exhibits clear host‐associated genomic diversification. Phylogenomic analyses have identified two major lineages corresponding to host specialization: WDV‐W, infecting wheat, and WDV‐B, infecting barley. Within these groups, isolates can be further subdivided into six clades (A–F), with clades A and F predominating in barley and clades B–E more frequently associated with wheat (Figure 2b). An alternative classification similarly distinguishes WDV‐W (clades WDV‐A and WDV‐B) from WDV‐B groups (WDV‐A1, WDV‐A2, WDV‐Bar (TR)) (Wu et al. 2015; Pfrieme et al. 2023). Host specificity appears to be mediated largely by sequence divergence within MP and non‐coding regulatory regions, which influence viral movement and replication efficiency in different *Poaceae* hosts.

The viral CP, responsible for encapsidation and vector interaction, displays additional structural and functional variation across clades, supporting a model of co‐evolution between virus, host, and vector. These genomic features collectively underpin the virus's epidemiology, determining host range, replication dynamics, and transmission efficiency. Wild and cultivated grasses serve as natural reservoirs that maintain viral diversity and facilitate inter‐species transfer across agricultural landscapes (Vacke 1972; Wu et al. 2015; Hassan et al. 2017; Abt et al. 2020; Pfrieme et al. 2023). The compact but functionally sophisticated genome highlights WDV's evolutionary optimisation for exploiting host cellular machinery while evading defence responses.

Taken together, WDV genome architecture can be viewed as a set of functional modules that converge on a common outcome that involves reprogramming host physiology to favour replication and systemic spread. Rep/RepA provide the central regulatory axis linking genome amplification to host transcriptional and cell‐cycle control, whereas MP/CP shape tissue tropism, long‐distance movement, and the efficiency of vector‐mediated transmission. Variation within MP and non‐coding regulatory regions likely tunes replication–movement trade‐offs across host backgrounds and environments, offering a mechanistic bridge between strain diversity and multi‐stress outcomes observed in the field. Viewed this way, WDV genome structure is relevant not only for taxonomy and strain biology but also for identifying the viral modules most likely to drive multi‐stress reprogramming in the host.

## 6 Replication Strategy and Early Host Reprogramming

WDV replication is not only a virological process but also a trigger for host‐scale reprogramming. By forcing access to replication machinery, altering transcriptional states, and interfering with defence‐linked pathways, replication‐associated functions create the permissive conditions that later shape hormone balance, symptom development, and stress integration.

In nature, WDV infection is initiated when viruliferous *Psammotettix alienus* injects saliva containing virions into phloem tissues during feeding (Wang et al. 2014). Although agroinoculation and biolistic delivery can experimentally introduce the virus, these artificial methods are less efficient and often fail to replicate the precise infection dynamics seen in vector‐mediated inoculation (Cejnar et al. 2019).

Following entry, virions traffic to the nucleus where replication proceeds via rolling‐circle replication (RCR), a canonical mechanism among Geminiviruses. The single‐stranded viral DNA (ssDNA) is first converted into a double‐stranded replicative form (dsDNA) by host DNA polymerases, after which Rep and RepA orchestrate replication initiation at the conserved nonanucleotide motif within the LIR. Rep, the most functionally versatile protein encoded by WDV, introduces a site‐specific nick to initiate replication, recruits host replication factors, and modulates viral transcription. Its multifunctionality is underscored by its ability to suppress host gene silencing pathways and to regulate other viral genes, enabling efficient genome amplification and systemic infection (Mahmood et al. 2025; Ruhel and Chakraborty 2019; Settlage et al. 2005).

To establish a permissive environment for replication, WDV manipulates the host cell cycle. Like other Geminiviruses, it reprograms differentiated cells to re‐enter S‐phase, thereby providing access to nucleotide pools and replication machinery not normally available in mature plant tissues (De Beeck and Caillet‐Fauquet 1997; Reed et al. 2004; Shakir et al. 2023). In begomoviruses (genus *Begomovirus*, family *Geminiviridae*), a key mechanism underlying host cell‐cycle manipulation involves the replication‐associated protein Rep, commonly referred to as AL1, interacting with plant retinoblastoma‐related proteins (pRBR). Notably, AL1 binds pRBR via a domain distinct from the canonical LxCxE motif, and mutations that weaken this interaction markedly reduce viral DNA accumulation and restrict infection largely to vascular tissues (Kong et al. 2000). Because WDV belongs to the genus *Mastrevirus* and does not encode an AL1 gene per se, any analogous pRBR‐targeting function in WDV is likely mediated by Rep and/or RepA, while other viral proteins, including MP, may contribute indirectly to tissue tropism and symptom development (Xie et al. 1995; Shakir et al. 2023). These examples illustrate how specific host–virus protein interactions can shape tissue tropism and symptom severity.

Beyond cell cycle manipulation, WDV alters host metabolism, transcription, and hormone signalling. It disrupts chloroplast‐associated gene expression, remodels carbohydrate partitioning, and influences the accumulation of reactive oxygen species, which collectively contribute to dwarfism, chlorosis, and reduced vigour in infected plants (Jiang and Zhou 2023). Plants counter these effects through layered defence systems, including transcriptional gene silencing via DNA methylation, post‐transcriptional RNA silencing, miRNA‐regulated antiviral pathways, autophagy, hormone‐mediated signalling, and activation of resistance (R) genes (Gupta et al. 2021). However, viral suppressors encoded by WDV attenuate many of these responses, permitting systemic colonisation.

Recent transcriptomic analyses of WDV‐infected wheat highlight pronounced perturbations in auxin (AUX), cytokinin (CKs), brassinosteroids (BRs), ethylene (ET), abscisic acid (ABA), and gibberellin (GA) signalling pathways, with clear implications for both defence and development (Liu, Zhang, et al. 2020). For instance, repression of GA biosynthesis through downregulation of KSL3 contributes directly to the characteristic dwarf phenotype, while altered AUX transport and CKs dynamics further destabilise growth‐regulating networks (Wu et al. 2017). The findings underscore how WDV exploits hormone crosstalk and transcriptional reprogramming to reshape host physiology in ways that reinforce viral success.

These replication‐linked processes therefore serve as the mechanistic bridge between viral genome function and the broader signalling disturbances that become visible at hormonal, physiological, and ecological scales.

## 7 Symptom Development as an Emergent Output of Host Reprogramming

In a concept‐driven view of WDV pathogenesis, symptoms should not be interpreted merely as visible consequences of infection. Instead, dwarfing, chlorosis, and heading defects can be understood as emergent outputs of disrupted coordination among developmental, metabolic, redox, and defence‐related pathways.

WDV infection produces characteristic physiological and morphological alterations, including severe dwarfing, chlorosis, and a pronounced reduction or complete absence of heading (Lemmetty and Huusela‐Veistola 2005). Symptom severity is strongly dependent on the developmental stage at infection: plants inoculated during early seedling growth exhibit profound stunting and impaired winter hardiness, whereas infections occurring after stem elongation generally cause only moderate shortening (Lindblad and Waern 2002; Pfrieme et al. 2024). These phenotypes arise from a complex interplay of viral replication, host transcriptional reprogramming, hormone imbalance, and metabolic disruption.

At the cellular level, WDV interferes with chloroplast integrity, represses photosynthesis‐associated genes, and perturbs carbohydrate metabolism, leading to impaired source‐sink relationships and energy deficits (Jiang and Zhou 2023). Simultaneously, viral manipulation of hormonal networks, including AUX, CKs, ABA, GA, BRs, and ET, creates widespread developmental dysregulation, which contributes directly to stunting and chlorosis (Liu, Zhang, et al. 2020).

Plants mount multilayered defence responses to WDV. RNA silencing forms the primary antiviral barrier, with transcriptional gene silencing (TGS) mediated by DNA methylation and post‐transcriptional gene silencing (PTGS) degrading viral RNA intermediates. Small interfering RNAs (siRNAs) and microRNAs modulate antiviral gene expression, while autophagy, ubiquitination pathways, and core protein kinases further support host resistance. However, Geminiviruses (including WDV) encode multiple suppressors that counteract silencing and destabilise immune signalling, enabling systemic viral movement and symptom progression (Hanley‐Bowdoin et al. 2013; Gupta et al. 2021).

This molecular tug‐of‐war between plant defence and viral suppression underlies symptom development. For example, Geminivirus‐driven S‐phase re‐entry via pRBR targeting (classically described for Begomovirus Rep/AL1) illustrates how cell‐cycle manipulation can expand viral tropism and intensify disease severity (Kong et al. 2000). Similarly, transcriptomic evidence shows that WDV alters ROS signalling and antioxidant systems, contributing to oxidative stress and tissue degeneration (Liu, Liu, et al. 2020; Kumar et al. 2021). Together, these processes construct the physiological landscape in which infection develops, linking molecular perturbations to whole‐plant phenotypes.

WDV symptom severity can be interpreted as an emergent readout of the host signalling hub. Replication‐linked cell‐cycle forcing, transcriptional and hormonal rewiring, and redox imbalance collectively determine the balance between growth maintenance and defence. The same network‐level perturbations that drive dwarfing and chlorosis (hormone imbalance, source‐sink disruption, ROS accumulation) also reshape the effectiveness of RNA silencing and other defence layers, thereby feeding back on viral titre and systemic movement. This framing helps explain why the timing of infection and host genotype strongly modulate both symptom development and resilience under additional abiotic or biotic pressures.

## 8 A Framework for Hormonal Crosstalk During WDV Infection

**FIGURE 3:**
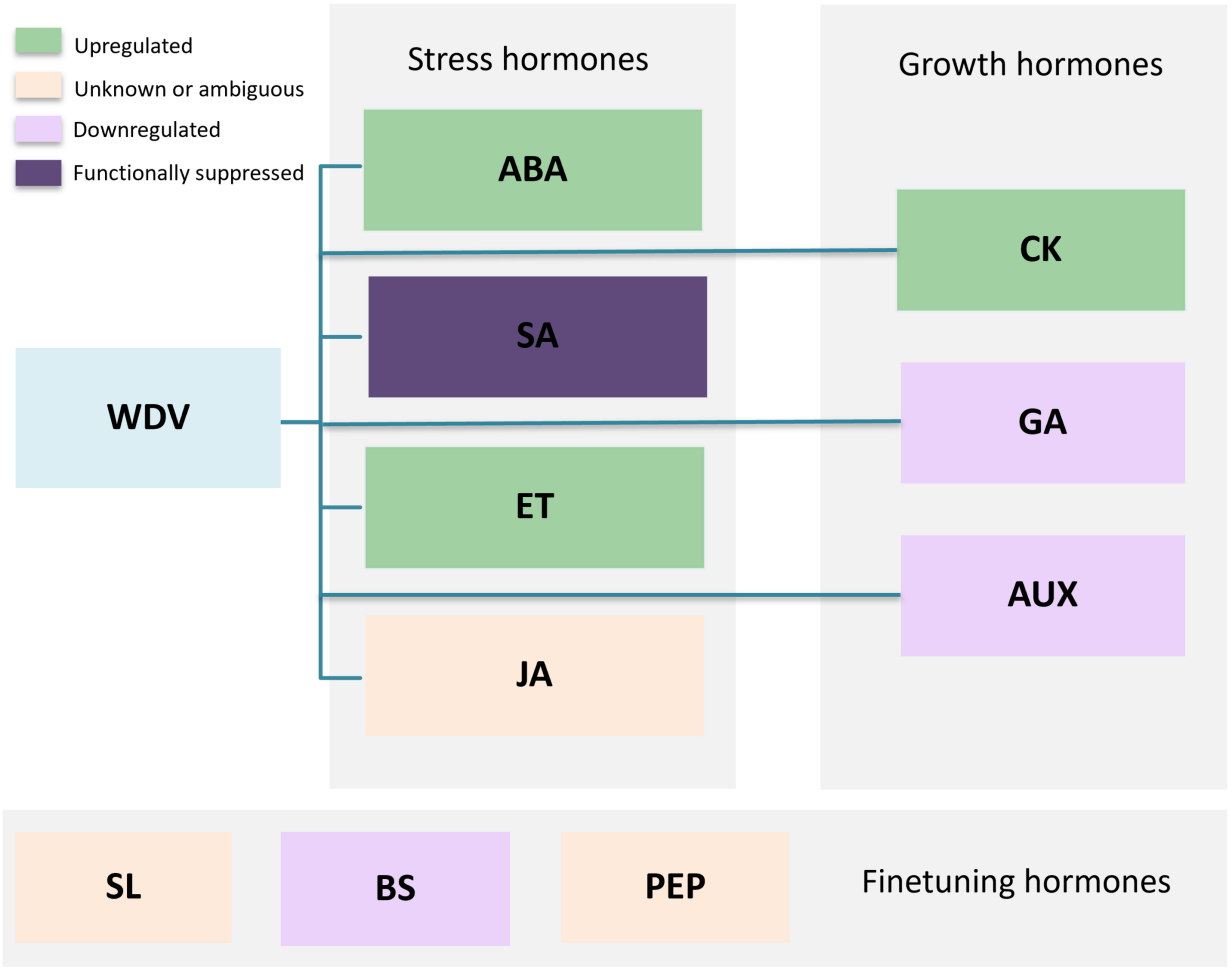
WDV alters wheat hormonal signalling by up‐regulating abscisic acid (ABA), ethylene (ET), and cytokinin (CK) pathways, while down‐regulating auxin (AUX), gibberellin (GA), and brassinosteroid (BR) metabolism. Salicylic acid (SA) signalling is functionally suppressed, and responses of jasmonic acid (JA), strigolactones (SL), and peptide hormones (PEP) remain context‐dependent. Colour coding indicates transcriptional trends derived from transcriptomic and physiological evidence (Liu, Liu, et al. 2020; Munné‐Bosch 2025).

Plant hormonal signalling is organised into a hierarchical regulatory architecture that coordinates development, stress perception and immune responses. This hierarchy can be conceptualised in three functional tiers. Tier 1 (growth hormones), including AUX, CKs and GA, regulate core processes such as cell division, elongation and organ patterning. Tier 2 (stress hormones), such as ABA, salicylic acid (SA), jasmonic acid (JA) and ET, govern biotic and abiotic stress signalling. Tier 3 (fine‐tuning hormones), including BRs, peptide hormones and strigolactones (SLs), fine‐tune systemic adjustments and maintain physiological homeostasis (Munné‐Bosch 2025). This hierarchical organisation provides a useful framework for visualising how WDV perturbs hormonal networks across stress‐ and growth‐related pathways (Figure 3).

Figure 3 is intended not only as a summary of hormone‐specific responses but also as a conceptual model showing how WDV shifts signalling priorities across a hierarchical regulatory system. This framing highlights how growth‐promoting, stress‐associated, and fine‐tuning hormones are reweighted rather than independently altered during infection.

WDV infection results in pronounced disturbances within this hierarchical system. Transcriptomic data indicate that Tier 1 pathways are significantly affected, with altered expression of genes involved in AUX transport and biosynthesis, as well as suppression of GA metabolism through downregulation of KSL3 (Wu et al. 2017; Liu, Liu, et al. 2020). Reduction of GA content contributes to the characteristic dwarf phenotype and disrupts developmental programming. AUX‐related disturbances may similarly affect root architecture, vascular differentiation and tillering, thereby exacerbating growth impairment. Tier 2 hormones are also influenced by WDV infection, although their disruption is more closely tied to stress signalling than to developmental architecture. ABA‐related transcripts often show activation, reflecting stress‐mimicking states and altered redox balance. In contrast, the involvement of SA and JA signalling in WDV responses remains unclear, with available transcriptomic data indicating variable or context‐dependent regulation rather than a consistent activation pattern (Liu, Liu, et al. 2020). These changes suggest a shift in the balance between stress adaptation and antiviral signalling. Although direct evidence is limited, Tier 3 hormone pathways may contribute to the broader physiological adjustments induced by WDV. Strigolactones, BRs and peptide hormones influence redox homeostasis, root symbioses, photosynthetic function and immune coordination (Francis et al. 2024; Soliman et al. 2022; Datta et al. 2024; Siddiqi and Husen 2021; Mansoor et al. 2024). Perturbations in lower tiers may propagate upward or fine‐tune regulatory processes in higher tiers, influencing the plant's capacity to maintain systemic stability during infection. Overall, transcriptomic profiles indicate that genes associated with AUX and BRs signalling and biosynthesis are predominantly downregulated, whereas those linked to ABA, ET, and CKs pathways tend to be upregulated in WDV‐infected wheat (Liu, Liu, et al. 2020). Reactive oxygen species couple hormonal pathways (ABA, SA, and JA) with environmental and pathogen‐derived cues. Although direct evidence from WDV remains limited, altered ROS dynamics likely contribute to broader hormonal reprogramming during infection (Fu and Dong 2013; Alazem and Lin 2015; Rahman et al. 2021).

WDV‐induced hormonal shifts are best understood as a structured reweighting of signalling priorities across the three‐tier hierarchy: growth‐promoting programmes (Tier 1) are suppressed while stress‐associated signalling (Tier 2) is favoured, with Tier 3 pathways potentially fine‐tuning systemic stability. Because ROS act as integrative signals that both regulate and are regulated by multiple hormones, redox imbalance provides a plausible coupling mechanism linking replication‐associated host reprogramming to hormone‐driven stress phenotypes. This tier‐based framework therefore converts descriptive transcriptomic patterns into a predictive model for how WDV can alter wheat performance under combined stresses. The main hormonal interactions, WDV‐induced effects, and possible functional consequences are summarised in Table 1.

**TABLE 1:** Hormonal crosstalk dynamics and functional consequences of Wheat Dwarf Virus (WDV) infection in wheat.

Hormone	Crosstalk partners	Main roles under normal conditions	WDV‐induced effect	Possible functional consequence
ABA	SA, JA, BR, (Li et al. 2022; Sharma et al. 2023; Wang et al. 2020)	Drought response, stomatal closure, osmotic regulation (Lee and Luan 2012; Lim et al. 2015)	↑ ABA activates drought‐type stress responses, represses SA defence (Liu, Zhang, et al. 2020; Rahman et al. 2021; Westwood et al. 2013)	Stomatal closure, enhanced stress mimicry (Lim et al. 2015; Liu, Liu, et al. 2020)
SA	JA, ET, ABA (Sharma et al. 2023; Shaukat et al. 2022)	Systemic acquired resistance (SAR), PR proteins (Ascencio‐Ibáñez et al. 2008; Gupta et al. 2021)	SA‐mediated defence may be functionally suppressed by ABA as in abiotic stress reaction (Sharma et al. 2023)	Reduced PR gene expression and weakened SAR due to ABA antagonism of SA signalling (Ascencio‐Ibáñez et al. 2008; Gupta et al. 2021; Sharma et al. 2023)
JA	SA, ET, GA, AUX, ABA (Li et al. 2022; Rehman et al. 2023; Wang et al. 2020)	Defence vs. necrotrophs, wounding, viruses (Pandita 2022; Singh et al. 2023)	No WDV‐specific evidence	Improved plant resilience by activating antioxidant systems and defensive pathway (Ali and Baek 2020; Wang et al. 2020)
ET	JA, SA, SL (Amist and Singh 2024; Naseer et al. 2024; Pérez‐Llorca et al. 2023)	Stress signalling, senescence (Das et al. 2015; Husain et al. 2020)	No WDV‐specific evidence	Possible impaired defence coordination (Francini and Ferrante 2023; Matilla‐Vázquez and Matilla 2014)
GA	ABA, JA, AUX, SL (Amist and Singh 2024; Banerjee and Roychoudhury 2019; Hou et al. 2013; Naseer et al. 2024; Yang et al. 2013)	Stem elongation, seed germination, flowering (Lupepsa et al. 2024)	↓ GA biosynthesis via KSL3 downregulation (Wu et al. 2017)	Dwarfism, growth inhibition (Kosakivska et al. 2022; Wu et al. 2017)
AUX	CK, ABA, SA (Joshi et al. 2023; Mallick et al. 2022; Nishiyama et al. 2011; Park 2007)	Growth, root/shoot patterning, vascular tissue formation, coordinating stress perception (Gurme et al. 2021; Jing et al. 2023; Park 2007)	Alteration of AUX transport and biosynthesis (Liu, Liu, et al. 2020)	Possible altered growth and root architecture (Salehin 2024)
CKs	AUX, ABA (Cortleven et al. 2019; Joshi et al. 2023; Liu, Zhang, et al. 2020; Nishiyama et al. 2011; Singh and Roychoudhury 2021)	Cell division, delay of senescence (Cortleven et al. 2019; Joshi et al. 2023; Liu, Zhang, et al. 2020; Nishiyama et al. 2011; Singh and Roychoudhury 2021)	↑ CKs may compensate for suppressed growth (Joshi et al. 2023; Singh and Roychoudhury 2021)	Partial cell division recovery, increased membrane stability, ABA reducing (Joshi et al. 2023; Liu, Zhang, et al. 2020; Nishiyama et al. 2011; Singh and Roychoudhury 2021)
BRs	ABA, AUX (Djemal et al. 2023; Rana and Hardtke 2020; Wang et al. 2020; Xuan and Beeckman 2021)	Growth promotion, stress response (Sharma et al. 2022; Siddiqi and Husen 2021)	No WDV‐specific evidence	Dependent on wide array of factors (Dehghanian et al. 2021; Yao et al. 2023)
SLs	ABA, CKs, GA, AUX, JA, SA, BR, ET (Amist and Singh 2024; Naseer et al. 2024)	Root development, mycorrhizal signalling (Sharma et al. 2024; Soliman et al. 2022)	Possible SL upregulation (Francis et al. 2024; Mansoor et al. 2024)	Improved nutrient uptake, ROS control under stress (Francis et al. 2024; Mansoor et al. 2024)
PEP	Unspecified	Cell‐to‐cell signalling, defence against microbial invasion, stress response modulation (Chen et al. 2020; Liu et al. 2025; Ortiz‐Morea and Reyes‐Bermudez 2019; Ren et al. 2024)	Possibly ↑ Peptide signalling (Liu et al. 2025; Ortiz‐Morea and Reyes‐Bermudez 2019)	Feasible enhanced immune response, systemic signalling, and hormone regulation (Liu et al. 2025; Manivannan and Umadevi 2019; Ortiz‐Morea and Reyes‐Bermudez 2019)

### 8.1 Mechanisms Linking Hormonal Crosstalk, ROS, and Antiviral Defence

Interactions among phytohormones play a decisive role in determining defence outcomes during WDV infection. These interactions operate through antagonistic, synergistic and hierarchical relationships that shape how plants prioritise responses under combined biotic and abiotic pressures. ABA frequently antagonises SA‐dependent antiviral signalling, resulting in weaker immune activation and stress states that resemble dehydration (Westwood et al. 2013; Checker et al. 2018; Liu, Liu, et al. 2020; Rahman et al. 2021). JA and SA are mutual antagonists that influence whether antiviral or anti‐herbivore pathways dominate, while ET interacts with both pathways to further modulate defence transcription (Alazem and Lin 2015; Islam et al. 2019; Pérez‐Llorca et al. 2023; Rahman et al. 2021; Zhao and Li 2021). GA, ABA and JA collectively shape growth defence trade‐offs. Suppression of GA biosynthesis during WDV infection promotes accumulation of DELLA proteins, which interact with JA signalling components and shift physiological priorities toward defence modulation and growth inhibition (Hou et al. 2013; Kosakivska et al. 2022; Wu et al. 2017). These processes form a signalling nexus that WDV exploits to suppress effective immunity while reinforcing the dwarf phenotype.

Reactive oxygen species (ROS) represent an additional point of integration within this crosstalk network. ROS activate redox‐sensitive transcription factors that regulate ABA‐, SA‐ and JA‐associated pathways, while hormones reciprocally control ROS production and scavenging systems. The hormone‐regulated control of siRNA and miRNA pathways further connects hormonal signalling to antiviral RNA silencing, which is central to host defence and strongly targeted by viral suppressors (Alazem and Lin 2015; Peighami Ashnaei 2025; Rahman et al. 2021).

These mechanistic interactions vary across wheat genotypes. Susceptible cultivars tend to activate multiple antagonistic hormonal pathways simultaneously, leading to diffuse and less effective defence responses. In contrast, resistant cultivars display more coordinated hormonal signalling, characterised by stronger SA‐based antiviral activation and precise temporal regulation of hormone crosstalk. This coordinated response is associated with milder disease symptoms and reduced viral titres, indicating the activation of effective host defence mechanisms rather than passive tolerance (Fraile and García‐Arenal 2010; Zhao and Li 2021; Sharaf et al. 2023). Together, these mechanisms illustrate how hormonal crosstalk determines host resilience or susceptibility under WDV infection. Crosstalk structures the balance between growth, stress adaptation and antiviral immunity and provides a mechanistic foundation for understanding symptom development, genotype‐specific responses and potential targets for improving resistance.

### 8.2 Hormone‐Specific Responses During WDV Infection

#### 8.2.1 Abscisic Acid (ABA)

ABA is a central regulator of drought tolerance, stomatal behaviour and osmotic adjustment, and its signalling intersects with immune pathways that shape responses to viral infection. Under WDV infection, ABA‐related genes are frequently upregulated (Liu, Liu, et al. 2020), consistent with infection‐induced stress states that mimic dehydration and oxidative imbalance. ABA promotes rapid stomatal closure, modifies membrane stability and enhances osmoprotective metabolites, which may contribute to the drought‐like phenotype often observed in infected cereals (Lee and Luan 2012; Lim et al. 2015). However, elevated ABA also suppresses SA‐mediated antiviral responses, weakening defence activation and potentially favouring viral accumulation. ABA further interacts with JA and ET signalling through shared transcriptional regulators and ROS‐dependent feedback loops (Fu and Dong 2013; Westwood et al. 2013; Wang et al. 2020; Rahman et al. 2021). These combined effects position ABA as a crucial mediator of WDV‐induced stress integration, simultaneously improving abiotic stress tolerance while dampening immune competence.

#### 8.2.2 Salicylic Acid (SA)

SA is central to antiviral defence, where it orchestrates the accumulation of pathogenesis‐related (PR) proteins, systemic acquired resistance (SAR) and R‐gene‐mediated responses (Ascencio‐Ibáñez et al. 2008; Gupta et al. 2021; Mishra et al. 2024). In WDV‐infected wheat, SA‐dependent processes appear suppressed or inconsistently activated, likely due to ABA–SA antagonism and virus‐induced transcriptional repression (Westwood et al. 2013; Liu, Liu, et al. 2020; Rahman et al. 2021). SA regulates catalase activity and modulates ROS homeostasis, producing controlled oxidative bursts required for antiviral signalling (Fu and Dong 2013; Rahman et al. 2021). Reduced SA activity may weaken these ROS‐dependent immune signals, contributing to systemic spread. Crosstalk with JA and ET further complicates the response, as SA typically suppresses JA‐dependent defences, thereby altering the balance between antiviral and anti‐necrotrophic pathways (Zhao and Li 2021; Pérez‐Llorca et al. 2023). The SA network therefore represents a critical vulnerability under WDV infection, where modulation of redox metabolism and hormone antagonism may jointly undermine resistance.

#### 8.2.3 Jasmonic Acid (JA)

JA regulates responses to wounding, herbivory, and necrotrophic pathogens, and cooperates with ET in several defence processes. Although JA‐specific data under WDV infection are limited, extensive knowledge from other viral systems informs plausible mechanisms. JA signalling interacts strongly with ROS dynamics and participates in balancing growth and defence, partly through its interaction with GA‐regulated DELLA proteins (Hou et al. 2013; Islam et al. 2019; Zhao and Li 2021). JA also influences antioxidant capacity and enhances the accumulation of secondary metabolites with protective functions (Ali and Baek 2020). Cross‐communication between JA and ABA shapes early defence responses, while antagonism with SA determines whether antiviral immunity or anti‐herbivore pathways dominate (Alazem and Lin 2015; Rahman et al. 2021; Zhao and Li 2021). These interactions suggest a role for JA in WDV symptom development and stress adaptation under combined abiotic stress.

#### 8.2.4 Ethylene (ET)

ET functions as a fast‐acting regulator of stress responses, senescence and defence signalling (Das et al. 2015). It interacts with JA in inducing defence transcripts and with SA in modulating PR‐gene expression (Pérez‐Llorca et al. 2023). Under viral infection, ET biosynthesis is often rapidly induced (Alazem and Lin 2015; Rahman et al. 2021), and transcriptomic patterns in WDV‐infected plants suggest ET pathway up‐regulation (Yu Liu et al., 2020). ET influences ROS production and scavenging, providing a regulatory feedback loop important for antiviral signalling. Its interactions with ABA and JA may further shape cell death, senescence and susceptibility, particularly during late infection stages (Rahman et al. 2021; Zhao and Li 2021).

#### 8.2.5 Gibberellins (GA)

GA signalling is one of the most strongly affected hormonal pathways under WDV infection. Experimental evidence shows that WDV suppresses GA biosynthesis by downregulating *ent*‐kaurene synthase‐like 3 (KSL3), resulting in a substantial reduction of GA_3_ levels and pronounced dwarfism, which can be reversed by exogenous GA treatment (Wu et al. 2017). The reduction of GA leads to DELLA protein accumulation, which restricts growth but also modulates immunity through interactions with JA signalling modules (Hou et al. 2013; Li et al. 2022). Transcriptomic analysis similarly reflects GA pathway suppression (Liu, Liu, et al. 2020). Together, these disturbances make GA a central determinant of symptom severity and support the view that WDV redirects plant resources from growth toward physiological states more favourable for viral replication.

#### 8.2.6 Cytokinins (CKs)

CKs regulate cell division, meristem identity and developmental patterning. In stress contexts, CKs promote membrane stability and delay senescence, with effects that counterbalance ABA‐mediated inhibition (Sharma and Prasad 2025). CKs pathway genes show mixed regulation in WDV‐infected wheat (Liu, Liu, et al. 2020), suggesting complex interactions between growth maintenance and stress response. Crosstalk between CKs, AUX and ABA is crucial in determining early defence dynamics, and their synergistic as well as antagonistic interactions, particularly in regulating meristem size under stress, are an active area of research (Mallick et al. 2022). Altered CKs homeostasis may reflect attempts to stabilise developmental processes disrupted by infection.

#### 8.2.7 Auxins (AUXs)

AUXs control virtually all aspects of morphogenesis, including stem elongation, root architecture and tropic responses (Gurme et al. 2021; Jing et al. 2023). WDV infection disrupts the expression of AUXs transporters and signalling factors such as AUX/IAA, GH3 and ARF genes. These disturbances may reduce vascular differentiation, inhibit tiller formation and contribute to the characteristic stunted phenotype. AUXs also interact with ROS signalling under biotic and abiotic stress. Crosstalk between AUXs, CKs, and ABA signalling networks may further influence meristem maintenance and stress adaptation, consistent with broader hormonal reprogramming observed during WDV infection (Liu, Zhang, et al. 2020; Rahman et al. 2021; Mallick et al. 2022).

#### 8.2.8 Brassinosteroids (BRs)

BRs regulate cell expansion, photosynthetic efficiency and tolerance to multiple abiotic stresses. Although BR‐specific data for WDV are limited, transcriptomic analyses indicate only subtle or partially altered expression of BR‐related genes in infected wheat rather than a consistent directional shift (Liu, Liu, et al. 2020). In other systems, BRs help regulate ROS homeostasis and modulate immune responses by enhancing antioxidant capacity and activating defence‐related genes (Nawaz et al. 2017; Siddiqi and Husen 2021; Sharma et al. 2022; Khajuria et al. 2022). BR pathways intersect with other hormonal networks, including ABA‐ and AUX/JA‐related signalling, suggesting a potential modulatory role in defence regulation under infection (Rana and Hardtke 2020; Wang et al. 2020; Xuan and Beeckman 2021; Djemal et al. 2023).

#### 8.2.9 Strigolactones (SLs)

In cereals, SLs inhibit axillary bud outgrowth and coordinate tiller formation, while also promoting mycorrhizal and other beneficial symbioses. Although no direct studies have examined SL function in WDV‐infected plants, SL signalling is tightly integrated with AUX, ABA and GA pathways that are perturbed during infection (Soliman et al. 2022; Naseer et al. 2024; Amist and Singh 2024). SLs additionally modulate ROS production and redox balance and contribute to whole‐plant stress adaptation and symbiotic competence (Francis et al. 2024; Mansoor et al. 2024; Sharma et al. 2024). Given their role in tillering and shoot architecture, SLs may contribute to tillering suppression during WDV infection, but this remains poorly explored.

#### 8.2.10 Peptide Hormones (PEP)

Peptide hormones and other small signalling peptides have emerged as important regulators of plant growth, stress adaptation and immunity (Chen et al. 2020; Ren et al. 2024). Antimicrobial and elicitor peptides, including defensins and other cysteine‐rich or post‐translationally modified peptides, contribute to defence against microbial pathogens and insect pests and can reinforce pattern‐triggered and effector‐triggered immunity (Manivannan and Umadevi 2019). Peptide elicitors (Peps) integrate environmental stress cues with phytohormone networks and ROS dynamics, engaging immune kinase cascades that intersect with ABA‐, SA‐ and JA‐dependent responses (Alazem and Lin 2015; Rahman et al. 2021; Liu et al. 2025). Although WDV‐specific data are not yet available, peptide hormones are likely to participate in early perception of infection, systemic signal propagation and fine‐tuning of antiviral defence, representing an important but still largely unexplored component of WDV–host interactions.

## 9 Microbiome Implications of WDV‐Driven Host Reweighting

Although no direct studies exist, WDV is likely to influence the wheat microbiome through changes in root exudation, hormonal signalling, and photosynthetic performance. Evidence from *Arabidopsis* demonstrates that pathogen infection triggers systemic release of microbe‐attracting compounds that recruit beneficial rhizosphere microbes capable of enhancing induced resistance or producing antimicrobial metabolites (Berendsen et al. 2012; Weller et al. 2012). Hormones disrupted by WDV (including AUX, CKs, and ABA) also regulate microbiome composition (Liu, Liu, et al. 2020). Understanding how WDV reshapes microbial communities could reveal new avenues for natural disease suppression.

### 9.1 Future Research Directions and Testable Predictions of the Hub Framework

Future work should not only expand the descriptive inventory of WDV‐associated responses but also test the central predictions of the host‐signalling‐hub framework. In particular, this framework predicts non‐additive outcomes under combined stress, strong dependence on genotype, developmental stage, and infection order, and disproportionate importance of key mechanistic nodes such as Rep/RepA‐linked host reprogramming, hormone–ROS coupling, and interference with RNA silencing. The priorities below provide practical routes for testing these predictions and translating the framework into agronomically relevant understanding.

One priority is the functional validation of host genes associated with resistance, where gene editing methods such as CRISPR/Cas9 and Virus‐Induced Gene Silencing (VIGS) can confirm the roles of candidates identified through transcriptomics. A second key need is the development of time‐resolved, integrated multi‐omics datasets that combine transcriptomic, proteomic, and metabolomic information. These approaches would help reveal regulatory pathways that operate during the earliest stages of infection as well as during systemic spread. Clarification of antiviral RNA interference processes is also required, particularly through small RNA sequencing and degradome analysis, to determine how WDV interferes with host gene‐silencing mechanisms. Another critical gap concerns interactions between WDV and major fungal pathogens, including species of *Fusarium*, *Zymoseptoria*, and *Puccinia*. Experimental studies on co‐infection could uncover synergistic or antagonistic effects with important agronomic consequences. Population‐level analyses such as genome‐wide association studies using diverse germplasm panels may also identify rare alleles contributing to tolerance. In addition, engineering of synthetic promoters that respond to multiple stresses might improve the precision of antiviral gene expression in field conditions. Research on the leafhopper vector *Psammotettix alienus*, particularly its genomics and behavioural responses, could reveal vulnerabilities in the transmission cycle. Finally, investigation of microbiome alterations in WDV‐infected plants may help identify beneficial microbial groups with potential for natural disease suppression. Together, these directions advance WDV research.

Collectively, these priorities shift the field from description to causality. They are designed to identify which hub edges are mechanistically decisive, including Rep/RepA‐linked cell‐cycle control, hormone–ROS coupling, and RNA silencing interference, and to determine how these nodes translate into agronomically relevant outcomes under co‐stress conditions. Resolving these links will be essential for moving from symptom‐based management to predictive, systems‐level strategies for resistance breeding and integrated control.

## 10 Conclusion

Wheat dwarf virus should be understood not simply as a phloem‐limited cereal pathogen, but as a systems‐level reprogrammer of host regulation. By altering cell‐cycle control, hormonal coordination, redox homeostasis, RNA silencing, and source‐sink balance, WDV reshapes how wheat integrates simultaneous biotic and abiotic cues. Viewing WDV through the host‐signalling‐hub framework helps explain why disease outcomes are strongly context‐dependent and why symptom severity, stress tolerance, and co‐infection dynamics vary across genotypes and developmental stages. This perspective also provides a more predictive basis for future research, resistance breeding, and integrated disease management under changing climatic conditions.

## Author Contributions

Jana Asszonyi conceived and designed the review, performed the literature search and data synthesis, prepared all figures and the summary table, drafted the manuscript, integrated feedback, and coordinated the preparation of the final version.

## Funding

This work was supported by Mendelova Univerzita v Brně (IGA26‐AF‐IP‐010, QL24010142).

## Conflicts of Interest

The author declares no conflicts of interest.

## Data Availability

Data sharing is not applicable to this article as no new data were generated or analysed in this review.
